# Beyond COVID-19: reconsidering routine contact precautions for common respiratory viruses

**DOI:** 10.1017/ash.2026.10835

**Published:** 2026-08-28

**Authors:** Gülsüm İclal Bayhan

**Affiliations:** Department of Pediatric Infectious Diseases, Faculty of Medicine, Ankara Yildirim Beyazit University, Ankara, Türkiye

Dear Editor,

I read with great interest the recent commentary by Jurica JM et al., which thoughtfully reexamines the continued use of contact precautions (CP) for patients with COVID-19.^1^ The authors highlight that, early in the pandemic, CP were appropriately implemented because the transmission dynamics of SARS-CoV-2 were poorly understood and fomite transmission was considered a plausible route. However, accumulating evidence from epidemiological investigations and experimental research has consistently demonstrated that airborne and droplet transmission are the predominant modes of spread, whereas the risk of fomite-mediated transmission is exceedingly low.^1,2^ Despite these data, CP have largely remained part of routine infection prevention policies in many healthcare institutions.^1^

While I agree with the authors’ conclusions regarding COVID-19, I believe that the same evidence-based approach should be applied to other common respiratory viruses for which routine CP continue to be recommended. Unlike SARS-CoV-2, these viruses have not been the focus of rigorous prospective studies designed to clarify their transmission dynamics in healthcare settings. The Centers for Disease Control and Prevention (CDC) still recommends CP for respiratory syncytial virus (RSV), human metapneumovirus (hMPV), and parainfluenza virus infections. However, these recommendations rely largely on historical infection prevention practices and indirect assumptions rather than contemporary evidence directly evaluating the contribution of contact transmission to nosocomial spread in healthcare settings.^3^

Experimental studies have demonstrated that RSV is transmitted predominantly through close contact with infectious secretions and subsequent self-inoculation of the nose or eyes after contact with contaminated surfaces, whereas small-particle aerosol transmission appears to play only a limited role.^4^ Accordingly, hand hygiene has long been regarded as probably the single most important procedure for preventing nosocomial RSV transmission. However, studies evaluating individual infection control measures have yielded conflicting results and have been insufficient to determine which combination of personal protective equipment (PPE) is most effective or evidence-based, leaving the independent contribution of gloves, gowns, and masks uncertain.^5^ In a multicenter study evaluating infection control practices for hospitalized children with RSV, considerable variation existed in isolation policies across participating hospitals. None of the centers fully adhered to CDC recommendations, and none required routine eye protection for healthcare workers (HCWs) as part of CP. Importantly, no isolation policy was associated with a reduction in nosocomial RSV transmission, whereas requiring all individuals entering the patient’s room to wear gowns was paradoxically associated with an increased risk of transmission.^6^ Consistent with these findings, a subsequent systematic review concluded that PPE strategies incorporating eye protection appeared to be more effective than those relying primarily on gowns and masks.^7^ Together, these findings highlight the limited evidence supporting the incremental benefit of individual CP components and underscore the need for high-quality contemporary studies to inform future recommendations.

Beyond their adverse effects on patient satisfaction, healthcare worker–patient interactions, healthcare costs, and environmental burden through increased plastic waste,^1^ routine contact precautions for common respiratory viruses may be difficult to implement consistently in real-world clinical practice, particularly in pediatric settings. During the autumn and winter respiratory virus season, pediatric emergency departments, outpatient clinics, and inpatient wards frequently become overwhelmed with children presenting with RSV and other viral respiratory infections. Under these conditions, maintaining strict adherence to CDC-recommended CP for every eligible patient becomes increasingly challenging, creating a substantial gap between written infection prevention policies and routine clinical practice.

Taken together, the available evidence suggests that recommendations for routine CP for common seasonal respiratory viruses should be re-evaluated using the same evidence-based principles recently applied to COVID-19. Future prospective studies are needed to identify which infection prevention measures provide meaningful protection and to support evidence-based updates of infection prevention guidelines.
